# Dual biotic stressors shape volatile organic compound emission patterns in pome fruit trees

**DOI:** 10.1080/15592324.2026.2700902

**Published:** 2026-07-09

**Authors:** Ali Karimi, Jannicke Gallinger, Jürgen Gross

**Affiliations:** a Institute for Plant Protection in Fruit Crops and Viticulture, Julius Kühn-Institut, Federal Research Institute for Cultivated Plants, Dossenheim, Germany; b Department of Crop Protection, Hochschule Geisenheim University, Geisenheim, Germany

**Keywords:** Herbivore-induced plant volatiles, plant-insect interactions, volatile organic compound, brown marmorated stink bug, apple proliferation, pear decline

## Abstract

Plants release volatile organic compounds (VOCs) as part of their defense mechanisms when attacked by phytopathogens and herbivores. These specific volatile emissions can help plants avoid damage. In this study, we investigated the effects of the brown marmorated stink bug (BMSB), *Halyomorpha halys*, and the phytopathogens “*Candidatus* phytoplasma mali”, causing apple proliferation (AP) or “*Ca*. P. pyri”, causing pear decline (PD) on the volatile emissions from apple (*Malus domestica*) or pear (*Pyrus communis*) trees. We analyzed VOCs emitted by uninfected and infected trees exposed to male and female BMSB. Using random forest analysis, the profiles of uninfected trees and those singly infected with PD- or AP-phytoplasma were clearly distinguishable from those infested with BMSB and double-infections with both phytoplasma and BMSB. BMSB-infested apple and pear trees emitted higher levels of linalool and 4,8-dimethyl-1(*E*)-3,7-nonatriene (DMNT) compared to uninfested trees. In addition, dual infections with phytoplasma and BMSB showed an induction in the content of these VOCs. Phytoplasma-infected apple trees significantly increased emissions of methyl salicylate (MeSA), whereas MeSA content was significantly increased in pear trees exposed to BMSB and also dual infections with phytoplasma and BMSB. In apple trees, *α*-farnesene was emitted at a higher amount only from phytoplasma-infected trees exposed to BMSB males, whereas in pear trees this VOC was significantly induced in response to female BMSB and dual infections with phytoplasma and female BMSB. Furthermore, we found three acids in healthy pear trees, *n*-hexadecanoic acid, octadecanoic acid, and oleic acid, which were not detected in phytoplasma-infected or BMSB-infested trees. It was demonstrated that this information can help to identify specific chemical signatures and specific VOCs, which may be used as biomarkers for detecting and monitoring these pests.

## 1. Introduction

When plants are exposed to biotic stress, such as insect feeding or pathogen attack, they adjust their defense responses through complex signaling networks that often include the emission of volatile organic compounds (VOCs).^1^ These VOCs can mediate direct and indirect defense mechanisms,^2^ act as signals for neighboring plants, attract natural enemies of herbivores, or inhibit pathogen growth.^3^ The composition and quantities of released VOCs can vary depending on stressors as well as plant species involved.^4^ Herbivores from different feeding guilds are often associated with the activation of distinct phytohormonal pathways, although these relationships are not universal and can vary across plant and insect species. Chewing insects, for example, tend to induce jasmonic acid (JA)-related responses, whereas piercing-sucking insects are discussed to activate JA-, salicylic acid (SA)-, or mixed signaling, depending on the feeding site and plant tissue targeted.^5^ ^,^ ^6^ In contrast, viruses and biotrophic pathogens are generally linked to SA-associated defenses.^7^ ^,^ ^8^

In pome fruit growing, phytoplasmas, cell-wall–lacking bacterial phytopathogens, are one of the most severe disease agents, which cause significant economic losses in pome fruit production. Pear decline (PD) and apple proliferation (AP) are destructive diseases of pear and apple trees caused by “*Candidatus* Phytoplasma pyri” and *“Candidatus* Phytoplasma mali”, respectively.^9^ ^,^ ^10^ These phytoplasmas colonize phloem tissue, where they impair assimilate transport and interfere with the plants hormonal balance, leading to characteristic symptoms such as leaf curling, premature leaf drop, reduced fruit quality, witches' broom, and stunted growth.^11^ These pathogens are transmitted by phloem-feeding psyllids: the pear psyllids *Cacopsylla pyri*, *C. pyricola*, and *C. pyrisuga* transmit “*Ca.* P. pyri”, while *C. picta* and *C. melanoneura* transmit “*Ca.* P. mali”.^12^ Phytoplasma infections are known to alter host volatile emission,^13^ potentially modifying the cues used by herbivores and vectors during host location.

While such interactions are predominantly investigated within pairwise systems, such as plant-pathogens, plant-arthropod, or arthropod-pathogens associations,^14^ natural systems are much more complex. Multiple organisms, such as plant pathogens and herbivorous insects, commonly co-infest the same host plants, and their combined effects can modify plant physiology in ways that extend beyond the classic pathogen-vector relationship. Therefore, the effects of plant-pathogen-herbivore interactions can play a major role in shaping chemical signaling.^15^ The VOC emissions from the same plant can be very specific to the individual pest or dual infestations with herbivores and phytopathogens.^16^ Importantly, even non-vector herbivores may interact with phytoplasma-infected trees in ways that alter or amplify volatile emissions, potentially generating pest- or stressor-specific VOC patterns.^17^

One abundant non-vector herbivore frequently attacking pome fruit is the brown marmorated stink bug (BMSB), *Halyomorpha halys*. This invasive pentatomid, native to East Asia, has become a major agricultural pest,^18^ which has spread rapidly and established on multiple continents, causing significant economic losses due to its feeding on a broad range of horticultural crops.^19^ Like other pentatomids, BMSB possesses piercing-sucking mouthparts to pierce food sources (e.g., xylem, phloem, and fruits) and obtain nutritional resources.^20^ Piercing-sucking insects can influence plant defense signaling pathways and the emission of VOCs by delivering substances such as digestive enzymes for tissue breakdown through their stylet canal.^6^ These interactions may induce JA, SA, or mixed JA-SA hormonal responses, depending on tissue specificity and feeding intensity, as shown for other piercing-sucking insects,^21^ ^,^ ^22^ which in responses ultimately shape the host plant's volatile blend. Despite the numerous studies on BMSB pheromone,^23^ ^,^ ^24^ few studies have investigated plant-derived volatiles in response to this pest. Peterson et al.^25^ showed that BMSB feeding and oviposition altered VOC emissions in a species-specific manner, reducing cis-3-hexenyl acetate in peach while increasing nerolidol in tree of heaven. Although the sex-specific impact of BMSB on the host plant's volatile induction remains an unexamined gap in the chemical ecology field, differential gender effects have been studied in related pentatomids; For instance, it is reported that adult females of *Nezara viridula* induce significantly higher quantities of plant volatile emissions than males.^26^

Understanding these specific volatile-mediated interactions is crucial for developing strategies to enhance plant resilience and manage pest and disease pressures effectively. In general, plants and biotic stressors such as herbivores and phytopathogens can signal to each other through the emission of volatile compounds, which play distinct roles in ecological interactions.^15^ Changes in the VOC patterns of host plants can lead us to develop management strategies, such as electronic nose sensors,^27^ which can open new avenues to improve the early detection and monitoring of different pests.

Although there are limited studies on the individual effects of BMSB infestation or phytoplasma infection on host plant VOCs, evaluating how these combined stress factors impact individual volatile compound levels is essential to identify distinct, pest-specific chemical responses. We assume that the selected target pest organisms (BMSB, and the phytoplasmas “*Ca.* P. mali” and “*Ca.* P. pyri”) individually might lead to emission of similar or specific VOC profiles in pome fruit trees. Moreover, we hypothesize that interactions between the selected may enhance VOC emissions and/or induce pest-specific VOCs in host plants. Therefore, this study aims to: 1) investigate the possible specific impact of the selected pests representing two contrasting modes of plant attack: piercing–sucking herbivory and phloem-limited bacterial infection in apple and pear trees and 2) identify pest-specific VOCs, which could serve as biomarkers for detecting and monitoring these pests.

## 2. Materials and methods

### 2.1. Plant material and phytoplasma inoculation

Potted apple trees (*Malus domestica* cv. “Karneval”) were grafted in August 2023 on clonal rootstock cv. “Bittenfelder”. Pear trees (*Pyrus communis* cv. “Williams Christ”) were grafted on the rootstock cv. “Kirchensaller Mostbirne”, grown at Julius Kühn-Institut (JKI), Institute for Plant Protection in Fruit Crops and Viticulture (Dossenheim, Germany). Half of the apple and pear rootstocks were grafted using buds from trees infected with the respective phytoplasma. The apple rootstocks were infected with a virulent strain of “*Ca*. P. mali” (strain 12/93b),^9^ while the pear trees were infected with “*Ca*. P. pyri” (strain PD).^28^ Both infected and healthy trees were cultivated under the same conditions in an insect-safe greenhouse. Six weeks before the experiment, the potted trees were transferred to a controlled greenhouse chamber for adaptation. All trees used in the experiment were kept under controlled conditions (humidity: 60% ± 10%; temperature: 24 ± 2 °C; day: 14 h, night: 10 h) throughout both the adaptation period and the experiment. To protect the trees from mildew, a fungicide was applied before they were transported to the controlled greenhouse chamber.

### 2.2. Verification of phytoplasma infection in plants

The phytoplasma infection status of the plants was verified using DNA extraction and polymerase chain reaction (PCR). Before grafting the trees and conducting the experiments, all trees were tested for phytoplasma infection as described in Gallinger et al.^29^ DNA was isolated from leaves using the method described by Doyle and Doyle.^30^ Following the DNA extraction, quantitative PCR (qPCR) was conducted for the generic detection of phytoplasma DNA with the Bio-Rad CFX96 Thermal Cycler (Bio-Rad Laboratories GmbH, Munich, Germany). The concentration of phytoplasma was automatically calculated based on the quantification cycle values. This was done using a cloned 16S rDNA gene standard, which ranged from 10^1^ to 10^9^ copies in the qPCR, along with the manufacturer's internal software.

### 2.3. Insect

Adult *Halyomorpha halys* (BMSB) used in this study originated from a wild population collected between May and August 2023 in the JKI experimental orchard in Dossenheim, Germany. A laboratory colony was established from eggs laid by field-collected adults and maintained in a climate chamber at 24 °C, 70% relative humidity, and a 16:8 h (L:D) photoperiod as described in Koßmann et al.^31^ Adults were reared in BugDorm cages (30 × 30 × 30 cm; 1:1 sex ratio) and fed *ad libitum* with green beans, tomatoes, and sunflower seeds. For the experiments, adults were kept in additional rearing cages under controlled conditions (25 °C, 60% RH, 16:8 h L:D) and starved for 24 h prior to testing.

### 2.4. Experimental set-ups

A greenhouse experiment was conducted using a factorial arrangement in a randomized complete block design to evaluate whether feeding with BMSB, infection with phytoplasma, or dual infestations with BMSB and phytoplasma induce volatile responses in potted apple and pear trees. All trees were placed within the same controlled-environment chamber six weeks before the headspace sampling. To account for potential spatial heterogeneity inside the chamber (e.g., light or airflow differences), the chamber was divided into three benches, which served as blocks. On each bench, one replicate of each treatment was located: 1) uninfested (BMSB) and uninfected (phytoplasma) control trees; 2) infested with male adult BMSB; 3) infested with female adult BMSB; 4) phytoplasma-infected trees; 5) phytoplasma-infected trees infested with male adult BMSB; 6) phytoplasma-infected trees infested with female adult BMSB (*n* = 5 trees per treatment). To assess the feeding effects, we enclosed one branch per tree in a sleeve-net cage (40 cm × 20 cm). Five adult BMSB of the same sex (male or female) were released into each cage. During an exposure period of 24 h, the feeding activity was monitored three times per day by visually checking the position and behavior of the insects. In all cages, individuals were observed on the stem or leaves, indicating active feeding rather than resting on the netting. After 24 h, the insects and cages were removed, and headspace sampling was performed immediately from the same branch.

### 2.5. Headspace sampling

Volatile compounds released from apple and pear trees were sampled using a headspace collecting device (HSCD) as described by Karimi and Gross.^32^ The HSCD consisted of six individual odor collection systems connected to mass flow controllers, controlling flow rate and total collected air volume. To collect the volatiles, single branches of apple and pear trees were enclosed in polyethylene oven bags (Toppits, Melitta, Minden, Germany). Using closed-loop sampling mode, a stream of purified air (1 ln/min), was filtered through clean air filter cartridges (ICAF 2X6, Sigma Scientific, Micanopy, USA), and pumped through the oven bags until 30 L was reached. Volatiles were trapped on stainless-steel, pre-packed sample tubes with Tenax TA sorbent with a mesh size of 35/60 (Markes, Neu-Isenburg, Germany). The sampling tubes were sealed with Teflon-coated brass compression caps (Swagelok, PerkinElmer), and stored until analysis.

### 2.6. Volatile analysis using Thermodesorption-GC-MS

Before the TD-GC-MS analysis, 2 µL of ethyl dodecanoate dissolved in methanol (20 ng/µL) were injected onto each Tenax tube as an internal standard. The samples were analyzed using an automated thermal desorber (TurboMatrix™ ATD 650, PerkinElmer) connected to a gas chromatograph (Clarus® 680 GC, PerkinElmer) coupled with mass spectrometer (Clarus® 600 S, PerkinElmer) (GC-MS) as described previously.^32^ ^,^ ^33^ In brief, the details of thermal desorption process were as follows: sampled Tenax tubes were desorbed for 10 min at 250 °C and cold trap (Tenax TA) was held at −20 °C throughout the tube desorption process, then heated at a rate of 99 K/s to 250 °C and desorbed for 1 min. Following the desorption process, compounds were separated and detected using a GC-MS system. The GC was equipped with a non-polar Elite-5MS capillary column (30 m × 0.25 mm id × 0.25 µm film thickness, PerkinElmer). Helium was used as carrier gas. The oven temperature was programmed as follows: 40 °C for 1 min, heating from 50 to 180 °C at 5 K/min, and increased to the final temperature of 280 °C by a rate of 20 K/min, and held at 280 °C for 6 min. The GC inlet line temperature and the ion source temperature were set at 250 and 180 °C, respectively. The quadrupole mass detector was operated in the electron impact (EI) mode at 70 eV. All data were obtained by collecting the full-scan mass spectra within the range of 35–350 *m/z*.

### 2.7. Identification and quantification with AMDIS

The GC-MS chromatograms of analyzed samples were evaluated using “Automated Mass spectral Deconvolution and Identification System” (AMDIS, V. 2.71; National Institute of Standards and Technology NIST, Boulder, CO, USA) following the protocol in Gross et al.^34^ To identify volatiles, ion fragmentation patterns, retention times, and retention indices (RI) of detected compounds were compared with the standard compounds introduced in the same system according to previous studies.^33^ ^,^ ^34^ For compounds for which no standards were available, the RI values were obtained from NIST Chemistry WebBook, corresponding to the setup characteristics used (non-polar 5MS stationary phase material, column diameter, length, film thickness, carrier). Identification criteria for AMDIS were applied as follows: match factor ≥ 80% and the relative retention index deviation ≤ 5% from the reference value. For quantification, the deconvolution settings were set as follows: component width, 32; adjacent peak subtraction, one; resolution, low; sensitivity, medium; shape requirements, low; maximum penalty, 20; and signal-to-noise ratio, >50. Compared to the internal standard, the relative percentage composition of individual VOCs was calculated from the GC-MS peak areas.

### 2.8. Statistical analysis

All statistical analyzes were conducted in R statistical software (Version 4.3.0).^35^ To compare and visualize the overall differences in the volatile profiles of the samples, a supervised random forest (RF) approach was applied using the function randomForest from the package “randomForest” (version 4.7-1.2).^36^ The model was trained with 1500 trees (ntree = 1500), using the square root of the number of features (mtry = 8) at each split. Variable importance was calculated, and the proximity matrix was used for the visualization with multidimensional scaling plot (MDS). The importance of each compound was assessed using the Mean Decrease in Accuracy metric provided by the Random Forest model. Furthermore, the function ANOVA from the package “stats” (version 4.3.0)^35^ was used for the two-way analysis of variance (ANOVA) to examine the differences in the individual VOC levels between treatments for all extracted volatile compounds. Tukey's Honest Significant Difference (HSD) test (*p* ≤ 0.05) was used to compare the mean values of VOCs with significant differences across the treatments. The Bonferroni correction was performed to adjust for multiple tests.

Fold changes (log2) relative to the control treatment were calculated solely to visualize the strength of the effects and were not used for statistical testing. The log₂ fold change (log₂FC) for each VOC was calculated as follows:

$$\log\;₂\mathrm{FC}=\;\log\;₂\;\left(\frac{(x_{i}\;+\;0.0001)\;}{\left(\mu_{\mathrm{control}}\;+\;0.0001\right)}\right)\;$$

where *x* is the relative peak area of the VOC in sample *i*, and μ_control_​ is the mean relative peak area across all control replicates. A pseudocount of 0.0001 was added to avoid division by zero and to enable comparison of low-abundance compounds.

Pearson correlation analysis was performed using the function “rcorr” from the package “corrplot” (version 0.95)^37^ to measure the linear relationship between VOCs, BMSB and phytoplasma infection.

## 3. Results

### 3.1. Volatiles emitted by apple

**Figure 1.**
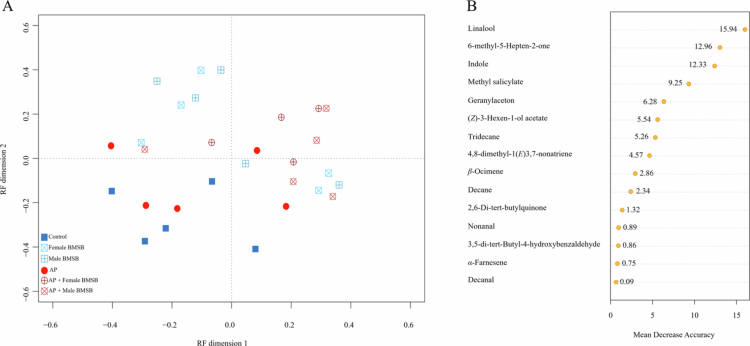
A) Multidimensional scaling (MDS) plot based on the random forest proximity matrix showing separation of VOC profiles among treatments in apple trees. Visualizing similarities and differences in overall VOC emission patterns for uninfested control trees, phytoplasma-infected trees (AP), male or female BMSB infestation (male BMSB, female BMSB), and dual infestations (AP + male BMSB, AP + female BMSB). B) The most discriminant volatiles based on mean decrease in accuracy following RF analysis of volatile profiles.

In total, sixty-eight volatile compounds have been detected and identified in the headspace of uninfected, phytoplasma-infected, or BMSB-exposed apple trees (Table 1 and Table S1A). The visualization through multidimensional scaling of the random forest proximity matrix revealed a clear separation of the VOC profiles from differently treated apple trees (Figure 1A). The confusion matrix shows that the majority of samples were correctly assigned to their respective treatment groups (Table S2). Specifically, uninfected and AP-phytoplasma-infected apple trees were clearly distinguishable from BMSB-infested and doubly infected apple trees (classification error: 0.2), whereas BMSB-only infestations were frequently misclassified between sexes (classification error: 0.4; Table S2, Figure 1A).

**Table 1.** Mean relative percentages of volatile compounds identified in headspace samples of control, phytoplasma-infected (AP), BMSB-infested (male or female), and dual-infested (AP and BMSB) apple trees.

Volatile organic compound^Ф^	RI^a^	Infested apple trees with BMSB			Infected apple trees with AP + BMSB^b^		
		Control	BMSB ♂	BMSB ♀	AP	AP + BMSB ♂	AP + BMSB ♀
Toluene	762	0.6	0.15	0.11	0.25	0.11	0.09
Hexanal^Ф^	801	0.38	1.21	1.05	0.43	0.51	0.91
(*E*)-2-Hexenal^Ф^	849	0.09	0.24	0.06	0.09	0.31	0.6
(*Z*)-3-Hexen-1-ol^Ф^	853	3.28	3.41	6.08	7.75	5.7	6.95
*m*-Xylene	867	0.52	0.21	0.17	0.42	0.18	0.16
1-Nonene	892	0.05	0.09	0.08	0.07	0.04	0.07
Heptanal^Ф^	902	tr	0.22	0.14	0.09	0.07	0.12
(*Z*)-2-Heptenal	955	nd	0.12	0.22	tr	0.11	0.14
Benzaldehyd^Ф^	958	0.56	0.57	0.51	0.55	0.27	0.17
6-methyl-5-Hepten-2-one^Ф^	987	0.11	0.67	0.64	0.42	0.49	0.74
Pseudocumene^Ф^	993	0.09	0.03	tr	0.05	tr	tr
Decane^Ф^	1000	0.28	0.29	0.23	0.21	0.15	0.15
Octanal^Ф^	1004	0.17	0.18	0.62	0.36	0.39	0.41
**(** ***Z*** **)-3-Hexen-1-ol acetate** ^**Ф**^	**1011**	**62.44** ^a^	**39.44** ^b^	**44.09** ^b^	**48.88** ab	**38.58** b	**46.02** b
4-Hexen-1-ol acetate	1014	1.31	0.82	0.58	1.18	0.46	0.89
Acetic acid hexyl ester^Ф^	1015	0.80	0.82	0.65	0.61	0.58	1.32
*p*-Cymene	1025	0.15	0.27	0.07	0.08	0.04	0.04
3-Cyclohexen-1-ol acetate	1027	1.19	0.43	0.36	0.36	0.19	0.58
Benzyl alcohol^Ф^	1034	0.12	0.21	0.25	0.23	0.21	0.28
Phenylacetaldehyd^Ф^	1043	0.15	0.34	0.18	0.27	0.08	0.10
*β*-Ocimene^Ф^	1049	0.32	1.01	1.33	0.21	0.68	0.76
Acetophenone^Ф^	1065	0.16	0.22	0.17	0.25	0.12	0.11
(*Z*)-Linalool oxide	1073	nd	tr	0.05	nd	nd	nd
(*E*)-Linalool oxide (furanoid)	1088	nd	0.06	0.11	nd	tr	tr
**Linalool** ^**Ф**^	**1099**	**tr**	**1.98** ab	**2.90** ^a^	**1.23** ^b^	**2.17** ab	**2.13** ab
Undecane^Ф^	1100	1.13	0.12	0.21	0.18	0.08	0.20
Nonanal^Ф^	1104	1.96	5.03	5.45	3.02	3.29	3.81
**4,8-Dimethyl-1(** ***E*** **),3,7-nonatriene** ^**Ф**^	**1117**	**1.44** b	**8.38** ^a^	**6.54** ab	**2.27** b	**5.50** ab	**2.95** ab
(*Z*)-3-Hexenyl butyrate^Ф^	1187	0.13	0.38	0.31	0.17	0.30	0.54
**Methyl salicylate** ^**Ф**^	**1194**	**4.62** ^b^	**5.93** ^b^	**3.91** ^b^	**12.60** ^a^	**8.62** ab	**8.24** ab
Dodecane^Ф^	1200	0.16	0.27	0.11	0.18	0.31	0.13
Decanal^Ф^	1206	1.46	4.11	4.61	2.60	2.32	3.13
(*Z*)-3-Hexenyl-*α*-methylbutyrate	1233	0.04	0.09	0.09	0.03	0.04	0.16
Nonanoic acid	1272	0.09	0.17	0.16	0.08	0.24	0.32
Indole	1294	tr	0.26	0.18	0.03	0.49	0.27
**Tridecane** ^**Ф**^	**1300**	**1.66** ^b^	**4.80** ^a^	**1.83** ^b^	**2.12** ^b^	**5.80** ^a^	**3.10** ab
Undecanal^Ф^	1307	0.16	0.39	0.37	0.25	0.20	0.25
Unknown01	1326	0.09	0.16	0.10	0.19	0.12	0.06
4,6-dimethyl-Dodecane	1327	0.09	0.04	0.04	0.07	0.05	tr
*α-*Cubebene	1352	0.03	tr	tr	tr	tr	tr
1-Tetradecane	1392	0.16	0.31	0.30	0.25	0.20	0.15
Tetradecane^Ф^	1400	0.15	0.17	0.07	0.18	0.13	0.08
Dodecanal	1410	0.13	0.26	0.25	0.18	0.10	0.16
*α*-Bergamotene	1417	nd	nd	0.03	nd	nd	0.03
*β*-Caryophyllene^Ф^	1423	1.28	1.60	1.70	0.78	1.75	2.36
*β*-Copaene	1432	0.08	0.03	0.05	0.03	tr	tr
Cadina-3,5-diene	1450	0.03	nd	tr	tr	nd	nd
**Geranylaceton** ^**Ф**^	**1454**	**0.29** ^b^	**1.63** ^a^	**1.26** ab	**1.09**ab	**1.07**ab	**1.71** ^a^
2,6,10-Trimethyltridecane	1463	0.06	0.06	0.03	0.05	0.03	tr
(*Z*)-Muurola-4(15),5-diene	1467	0.18	0.04	0.07	0.04	0.03	0.03
2,6-di-tert-Butylquinone	1469	0.82	0.54	0.32	0.63	0.39	0.26
*γ*-Muurolene	1480	0.04	0.03	0.03	tr	tr	0.05
Germacrene D	1485	0.21	0.07	0.10	0.04	0.08	0.10
(*Z*,*E*)-*α*-Farnesene	1496	0.40	0.37	0.27	0.11	0.66	0.23
Pentadecane^Ф^	1500	0.13	0.15	0.10	0.16	0.09	0.07
***α*** **-Farnesene**	**1510**	**4.64** ab	**4.89** ab	**4.86** ab	**2.76** ^b^	**11.68** ^a^	**4.07** ab
Calamenene	1527	0.13	0.05	tr	0.07	0.04	0.03
*α*-Cadinene	1542	0.03	nd	tr	nd	nd	nd
*α*-Calacorene	1547	0.05	tr	0.04	tr	tr	tr
(*Z*)-3-Hexen-1-ol benzoate^Ф^	1572	0.58	0.50	0.26	0.85	0.88	0.63
Hexyl benzoate	1580	tr	tr	tr	tr	0.06	0.03
Hexadecane^Ф^	1600	0.14	0.14	0.11	0.16	0.07	0.05
Hexanoic acid, 3,5,5-trimethyl-, 2-ethylhexyl ester	1658	0.12	0.42	0.15	0.22	0.22	0.09
Cadalene	1679	0.03	nd	tr	tr	tr	nd
3,5-di-tert-Butyl-4-hydroxybenzaldehyde	1772	0.61	0.79	0.70	0.49	0.38	0.37
Octadecane^Ф^	1800	0.06	0.07	0.03	0.06	0.04	tr
Nonadecane^Ф^	1900	0.04	0.05	tr	0.03	tr	nd
Hexadecanoic acid methyl ester	1930	0.07	0.18	0.03	0.12	0.07	0.17

For standard deviation values, see Supplementary Table S1A. tr, traces < 0.03%; nd, not detected.

^(Ф)^ VOCs identified based on the comparison of retention time and mass spectra data with an authentic standard.

^(a)^ RI, linear retention indices on nonpolar Elite-5MS column, experimentally determined using a homolog series of Kovats standard (C8-C20).

^(b)^ AP, infected apple trees with “*Ca*. P. mali”; ♀, female BMSB; ♂, male BMSB. The values are presented as means of 5 replicates. Statistically significant differences (adj. *p* ≤ 0.05) are indicated in bold. Different lowercase letters indicate significant differences in each row among treatments. All adjusted *p*-values are reported in Supplementary Table S3.

The out-of-bag (OOB) estimate of the error rate for the RF model was 24.14%. In addition, RF analysis highlighted 15 VOCs as discriminating between treatments. Among these VOCs identified by the model, linalool, 6-methyl-5-hepten-2-one, indole, and methyl salicylate (MeSA) exhibited the highest predictive importance with mean decrease accuracy (MDA) values exceeding 9 (Figure 1B).

**Figure 2.**
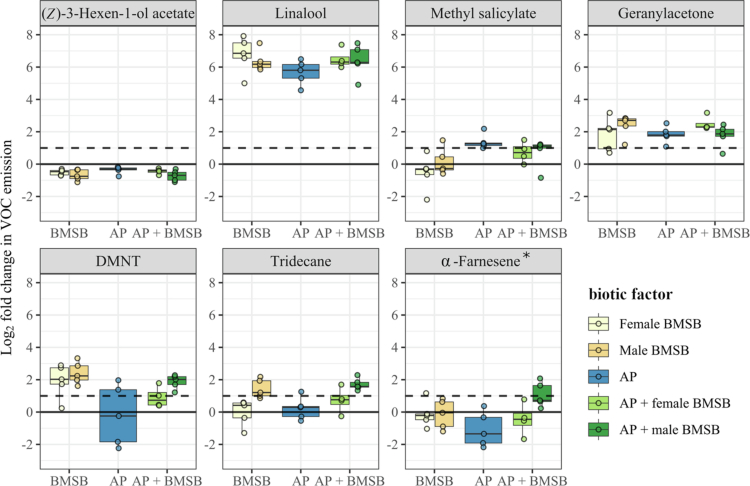
Log₂ fold-change of volatile organic compounds (VOCs) emitted by apple trees under different biotic stress treatments. Log₂ fold-changes were calculated for each VOC relative to the mean emission of control trees to visualize the direction and strength of treatment effects. Boxes represent the change in VOC emission in response to individual BMSB feeding (male or female), phytoplasma infection (AP), and dual infestations (AP + male BMSB; AP + female BMSB). Positive values indicate increased emission relative to controls, while negative values indicate reduced emission. The dashed line indicates a log₂ fold change of +1, corresponding to a twofold increase in VOC emission relative to control trees. *: *α*-farnesene identified based on the comparison of retention time and mass spectra data, whereas other VOCs identified using authentic standards.

A significant treatment effect was detected in the emission of seven VOCs emitted by uninfested apple trees compared to phytoplasma-affected, BMSB-infested, or dual-infested apple trees (Table S3). Methyl salicylate significantly increased only in AP-phytoplasma-infected trees (Tukey HSD, adj. *p* = 0.023), but not by BMSB-infestations (Figure 2). Linalool was detected only in trace amounts in VOC samples from uninfested trees, but it accounted for more than 1% of the relative emission in all other treatments (Figure 2). In contrast to MeSA, significant elevations of linalool were detected in response to infestations with male BMSB, female BMSB, and AP-phytoplasma in combination with BMSB feeding (adj. *p* < 0.05). Whereas DMNT was significantly enhanced by male BMSB feeding (adj. *p* = 0.005) (Figure 2). The amount of tridecane was increased after the branches were exposed to male BMSB exclusively (adj. *p* = 0.015) and simultaneous infestations with AP-phytoplasma and male BMSB (adj. *p* = 0.0009) (Figure 2). A moderate increase of geranyl acetone was detected for male BMSB and AP-phytoplasma and female BMSB infested apple trees (adj. *p* = 0.011 and 0.011) (Figure 2). While the ANOVA revealed significant main effects of AP-phytoplasma in combination with male BMSB (adj. *p* = 0.05) on the amount of *α*-farnesene in headspace samples of apples, post hoc Tukey tests with Bonferroni adjustment did not identify any significant pairwise differences (Figure 2). Emission of (*Z*)-3-hexen-1-ol acetate was significantly reduced in response to male and female BMSB feeding (adj. *p* = 0.001 and 0.012), as well as in AP-phytoplasma and BMSB dual infestations (male: adj. *p* = 0.0008; female: adj. *p* = 0.044), but not by single phytoplasma infection (adj. *p* = 0.100) (Figure 2).

**Figure 3.**
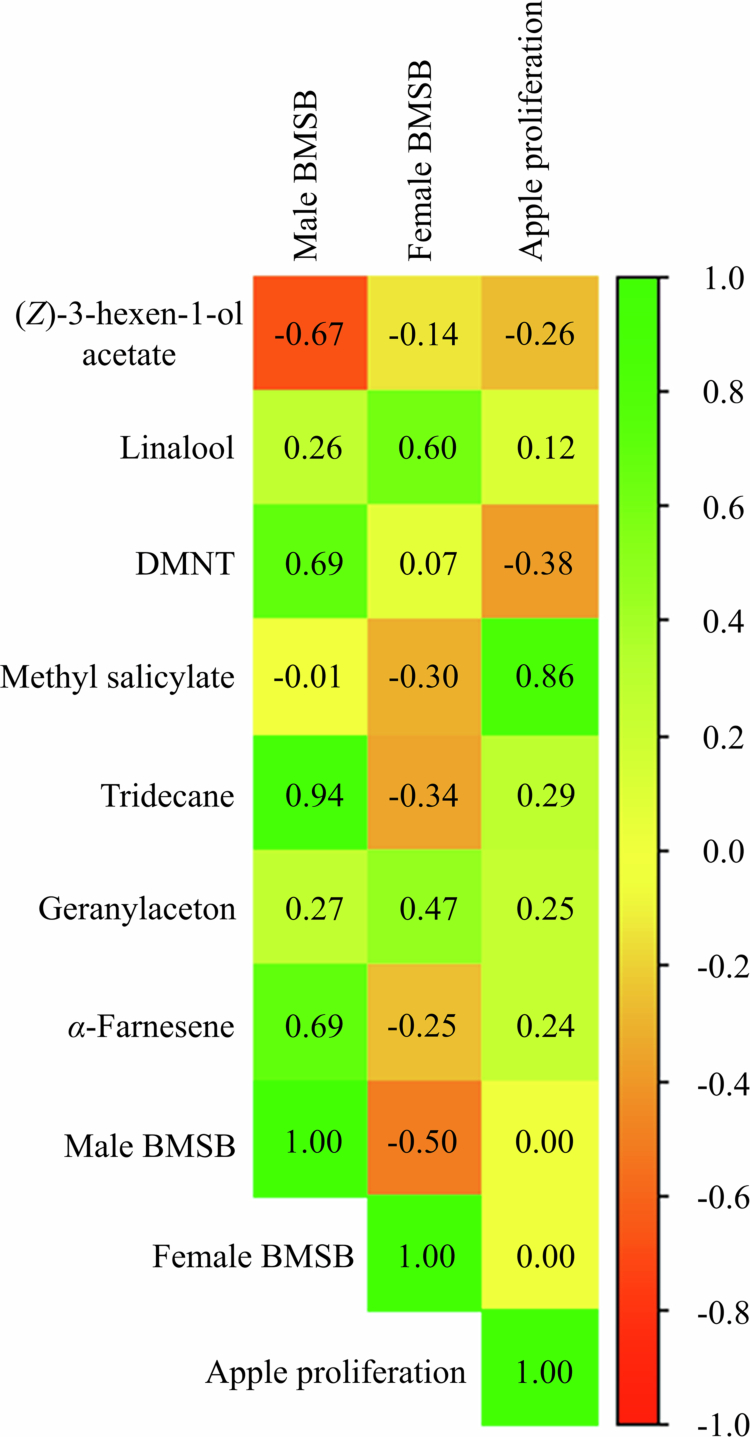
The correlogram represents the correlations between volatile compounds emitted by apple trees, BMSB feeding (male or female), and phytoplasma infection (AP). Positive correlations are displayed in green and negative correlations in red.

The correlation analysis (Figure 3) revealed that MeSA was the only compound positively correlated with apple proliferation infection (*r* = 0.86). Male BMSBs showed strong positive correlations with DMNT (*r* = 0.69), tridecane (*r* = 0.94), and *α*-farnesene (*r* = 0.69), while female BMSBs were positively correlated with linalool emission (*r* = 0.60).

### 3.2. Volatiles emitted by pear

**Figure 4.**
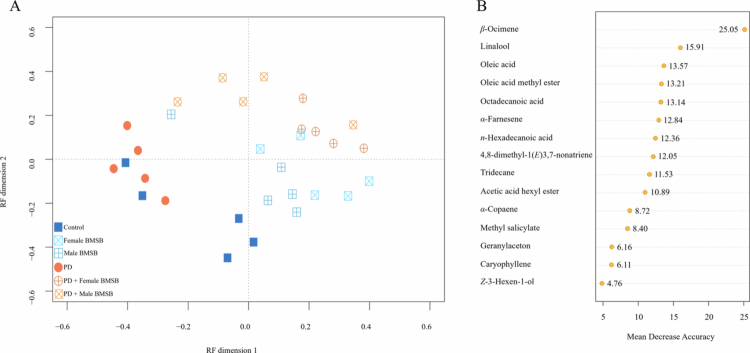
A) Multidimensional scaling (MDS) plot based on the random forest proximity matrix showing separation of VOC profiles among treatments in pear trees. Visualizing similarities and differences in overall VOC emission patterns for uninfested control trees, phytoplasma-infected trees (PD), male or female BMSB infestation (male BMSB, female BMSB), and dual infestations (PD + male BMSB, PD + female BMSB). B) The most discriminant volatiles based on mean decrease in accuracy following RF analysis of volatile profiles.

Seventy volatile compounds were detected and identified in the headspace samples of uninfected and phytoplasma-infected pear trees exposed to male and female BMSB (Table 2 and Table S1B). The RF analysis classified the samples into distinct groups based on their volatile profiles across the various treatments (Figure 4A). In addition, the confusion matrix (Table S4) provided a class-wise distribution of the model's predictive performance. Similar to apple trees, the profiles of uninfected pear trees and those singly infected with PD phytoplasma clustered together (within the resp. group) and distinguished clearly from those infested with BMSB and double-infections (classification error: 0.0, Table S4), while low misclassification rates were observed in BMSB-infested and double-infected trees (classification error: 0.2, Table S4). The out-of-bag (OOB) estimate of the error rate for the RF model was 6.67%. In view of the results from RF, the top 15 key volatile compounds were explored and considered as significant VOCs for separation between the treatments (Figure 4B). Among the key volatiles, *β*-ocimene, linalool, oleic acid, oleic acid methyl ester, and octadecanoic acid revealed the highest predictive importance with high MDA values.

**Table 2.** Mean relative percentages of volatile compounds identified in headspace samples of control, phytoplasma-infected (PD), BMSB-infested (male or female), and dual-infested (PD and BMSB) pear trees.

Volatile organic compound^Ф^	RI^a^	Infested pear trees with BMSB			Infected pear trees with PD + BMSB^b^		
		Control	BMSB ♂	BMSB ♀	PD	PD + BMSB ♂	PD + BMSB ♀
Hexanal^Ф^	801	0.41	0.61	0.75	0.29	0.52	0.91
(*E*)-2-Hexenal^Ф^	849	tr	0.13	0.12	0.07	0.14	0.25
(*Z*)-3-Hexen-1-ol^Ф^	853	5.19	5.20	4.65	12.19	12.25	9.03
*m*-Xylene	867	0.18	0.17	0.03	0.21	0.12	0.06
Heptanal^Ф^	902	0.15	0.08	0.09	0.07	0.10	0.16
(*E*,*E*)-2,4-Hexadienal	909	0.04	0.18	0.06	0.08	0.10	0.06
*p*-Benzoquinone	918	tr	0.04	nd	tr	0.03	nd
*α*-Pinene^Ф^	932	0.19	0.13	0.06	0.15	tr	0.06
(*Z*)-2-Heptenal	955	nd	0.06	0.14	nd	tr	0.21
Benzaldehyde^Ф^	958	0.51	0.48	tr	0.25	0.32	0.26
4-Oxohex-2-enal	959	0.21	1.09	0.48	0.39	0.55	0.46
6-methyl-5-Hepten-2-one^Ф^	987	0.54	0.28	0.46	0.25	0.14	0.24
*β*-Myrcene^Ф^	992	0.13	0.21	0.15	0.17	0.04	0.13
Decane^Ф^	1000	0.27	0.22	0.15	0.20	0.16	0.16
(*Z*)-3-Hexen-1-ol acetate^Ф^	1011	60.21	61.66	53.77	69.74	59.81	52.31
4-Hexen-1-ol acetate	1014	0.86	1.70	0.30	0.61	0.22	0.45
Acetic acid hexyl ester^Ф^	1015	0.45	0.13	0.19	0.33	0.93	0.86
Limonene^Ф^	1028	0.29	0.35	0.18	0.24	0.05	0.05
Benzyl alcohol^Ф^	1034	tr	tr	0.03	0.07	nd	tr
(*Z*)-*β*-Ocimene	1038	0.07	0.04	0.08	0.03	tr	0.03
***β*** **-Ocimene** ^**Ф**^	**1049**	**2.73** ^b^	**2.74** ^b^	**9.36** ^a^	**0.12**c	**1.52** ^b^	**3.69** ^b^
Acetophenone^Ф^	1065	0.20	0.20	0.14	0.11	0.17	0.18
Linalool oxide	1073	nd	tr	0.04	nd	nd	tr
(*E*)-Linalool oxide (furanoid)	1088	nd	0.04	0.24	nd	nd	tr
Methyl benzoate^Ф^	1095	0.16	0.13	tr	0.11	0.11	0.09
**Linalool** ^**Ф**^	**1099**	**nd**	**0.81** ab	**1.20** ^a^	**0.49** ab	**0.52** ab	**1.09** ^a^
Undecane^Ф^	1100	0.53	nd	nd	nd	nd	nd
Nonanal^Ф^	1104	2.79	3.75	4.20	2.34	3.15	3.90
**4,8-Dimethyl-1(** ***E*** **)3,7-nonatriene** ^**Ф**^	**1117**	**0.62** ^b^	**2.16** ab	**5.85** ^a^	**0.09** ^b^	**1.86** ab	**6.49** ^a^
Cosmene	1131	tr	tr	0.16	0.14	tr	0.07
(*Z*)-3-Hexenyl butyrate^Ф^	1187	nd	0.03	0.04	0.13	0.80	0.38
*α*-Terpineol^Ф^	1191	0.34	0.28	0.40	0.17	0.04	0.14
**Methyl salicylate** ^**Ф**^	**1194**	**0.14** ^b^	**2.34** ab	**3.39** ^a^	**0.07** ^b^	**2.86** ^a^	**3.23** ^a^
Decanal^Ф^	1206	2.28	1.70	1.93	1.53	2.01	1.99
(*Z*)-3-Hexenyl-*α*-methylbutyrate	1233	nd	tr	tr	0.03	0.15	0.15
Nonanoic acid	1272	0.24	0.23	0.32	0.20	0.31	0.37
**Tridecane** ^**Ф**^	**1300**	**0.31** ^b^	**0.49** ^b^	**1.01** ab	**0.30** ^b^	**1.97** ^a^	**0.96** ab
Undecanal^Ф^	1307	0.23	0.19	0.18	0.18	0.21	0.26
*α*-Cubebene	1352	nd	nd	nd	nd	tr	0.03
*α*-Copaene^Ф^	1379	0.24	0.38	0.28	0.08	0.29	0.38
1-Tetradecene	1392	0.37	0.38	0.24	0.29	0.28	0.28
*β*-Elemene	1394	tr	0.03	0.03	nd	tr	tr
Tetradecane^Ф^	1400	0.12	0.09	0.03	0.10	0.12	0.10
Dodecanal	1410	0.17	0.15	0.13	0.11	0.13	0.15
*β*-Caryophyllene^Ф^	1423	0.34	0.58	0.60	0.11	0.20	0.74
Geranylaceton^Ф^	1454	0.83	0.68	0.98	0.67	0.44	1.01
*α*-Hummulen^Ф^	1457	0.04	0.07	0.07	tr	tr	0.05
2,6,10-Trimethyltridecane	1463	0.03	tr	0.03	tr	0.03	0.03
*γ*-Muurolene	1467	nd	tr	0.03	nd	tr	0.03
Germacrene D	1485	nd	tr	0.04	nd	tr	0.03
(*Z*,*E*)-*α*-Farnesene	1496	nd	tr	0.05	nd	tr	0.04
*α*-Muurolene	1503	nd	0.03	0.04	nd	tr	0.03
***α*** **-Farnesene**	**1510**	**0.16** ^b^	**0.78** ab	**2.12** ^a^	**0.03** ^b^	**1.05** ab	**2.36** ^a^
2,4-di-tert-Butylphenol	1513	0.58	1.53	0.37	1.06	0.60	0.15
*δ*-Cadinene	1527	0.15	0.26	0.19	0.07	0.19	0.25
*α*-Calacorene	1547	tr	0.05	tr	tr	tr	0.04
*β*-Calacorene	1567	nd	0.03	tr	nd	0.03	tr
(*Z*)-3-Hexen-1-ol benzoate^Ф^	1572	0.04	nd	tr	tr	0.11	0.10
3E, 7E-4,8,12-Trimethyltrideca-1,3,7,11-tetraene	1581	tr	0.04	0.04	nd	0.06	0.15
Hexadecane^Ф^	1600	0.09	0.12	0.10	0.10	0.09	0.14
Cadalene	1679	nd	0.04	nd	nd	0.03	nd
Heptadecane^Ф^	1700	0.06	tr	0.03	0.11	0.05	0.05
3,5-di-tert-Butyl-4-hydroxybenzaldehyde	1772	0.65	tr	0.13	0.40	0.04	0.15
Octadecane^Ф^	1800	0.09	tr	tr	0.17	0.03	0.03
2-Ethylhexyl salicylate	1810	nd	0.03	nd	nd	0.04	nd
Hexadecanoic acid methyl ester	1930	0.16	0.11	0.09	0.13	0.13	0.09
***n*** **-Hexadecanoic acid**	**1968**	**0.86** ^a^	**nd**	**nd**	**nd**	**nd**	**nd**
Oleic acid methyl ester	2089	0.08	nd	nd	nd	nd	nd
**Oleic acid** ^**Ф**^	**2122**	**5.44** ^a^	**nd**	**nd**	**nd**	**nd**	**nd**
**Octadecanoic acid**	**2135**	**0.86** ^a^	**nd**	**nd**	**nd**	**nd**	**nd**

For standard deviation values, see Supplementary Table S1B. tr, traces < 0.03%; nd, not detected.

^(Ф)^ VOCs identified based on the comparison of retention time and mass spectra data with an authentic standard.

^(a)^ RI, linear retention indices on nonpolar Elite-5MS column, experimentally determined using a homolog series of Kovats standard (C8-C20).

^(b)^ PD, infected pear trees with “*Ca*. P. pyri”; ♀, female BMSB; ♂, male BMSB. The values are presented as means of 5 replicates. Statistically significant differences (adj. *p* ≤ 0.05) are indicated in bold. Different lowercase letters indicate significant differences in each row among treatments. All adjusted *p*-values are reported in Supplementary Table S5.

A significant treatment effect was detected in the emission of several VOCs emitted by uninfested pear trees compared with phytoplasma-affected, BMSB-infested, or dual-infested trees. No significant VOC differences were detectable in pear trees solely infected by PD phytoplasma compared to uninfested pear trees (Table S5).

**Figure 5.**
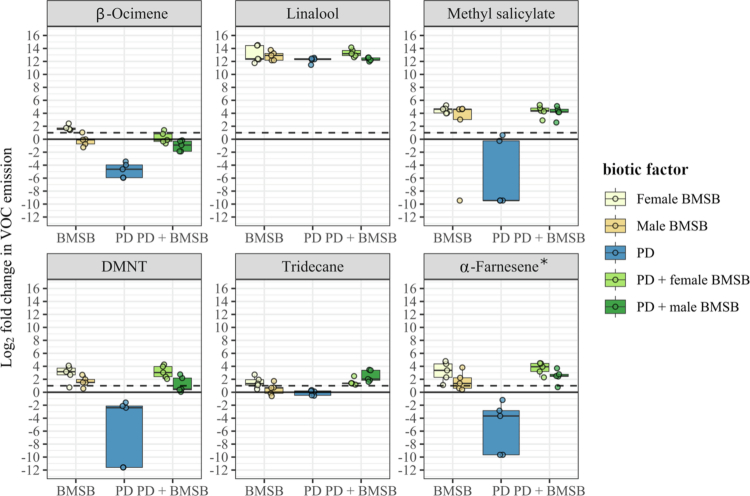
Log₂ fold-change of volatile organic compounds (VOCs) emitted by pear trees under different biotic stress treatments. Log₂ fold-changes were calculated for each VOC relative to the mean emission of control trees to visualize the direction and strength of treatment effects. Boxes represent the change in VOC emission in response to individual BMSB feeding (male or female), phytoplasma infection (PD), and dual infestations (PD + male BMSB; PD + female BMSB). Positive values indicate increased emission relative to controls, while negative values indicate reduced emission. The dashed line indicates a log₂ fold change of +1, corresponding to a twofold increase in VOC emission relative to control trees. *: *α*-farnesene identified based on the comparison of retention time and mass spectra data, whereas other VOCs were identified using authentic standards.

Methyl salicylate increased significantly in trees exposed to female BMSB (adj. *p* = 0.004), and also in PD-infected pear trees exposed to male and female BMSB (adj. *p* = 0.021 and 0.006, resp.) (Figure 5). PD alone did not affect MeSA levels (adj. *p* > 0.5). Tridecane emission was significantly elevated in phytoplasma-infected pear trees subjected to male BMSB feeding (adj. *p* = 0.002), but not in other treatment combinations. Linalool emission increased across all treatments (Figure 5), significantly elevated in response to female BMSB feeding without and in combination with PD-phytoplasma (adj. *p* = 0.008 and 0.017, resp.). Likewise, DMNT was significantly elevated in response to female BMSB feeding on pear trees without phytoplasma infection (adj. *p* = 0.023) as well as PD-phytoplasma-infected pear trees (adj. *p* = 0.009), whereas male BMSB and single phytoplasma infection did not induce significant changes (Figure 5).

The relative percentage of *β*-ocimene was significantly increased only in pear trees exposed to female BMSB (adj. *p* = 0.0001), while neither male BMSB nor phytoplasma infection alone significantly altered its levels (Figure 5). *α*-Farnesene was significantly increased in response to female BMSB feeding (adj. *p* = 0.043) and PD-infected pear trees exposed to female BMSB (adj. *p* = 0.017), whereas neither single PD infection nor male BMSB feeding altered its emission significantly (Figure 5). The green leaf volatile (*Z*)-3-hexen-1-ol acetate showed no significant treatment-related changes in any comparison (all adj. *p* > 0.28). Notably, three VOCs: octadecanoic acid, *n*-hexadecanoic acid, and oleic acid were detected only in healthy pear trees but were absent from all treatments involving BMSB, regardless of phytoplasma infection status (Table 2).

**Figure 6.**
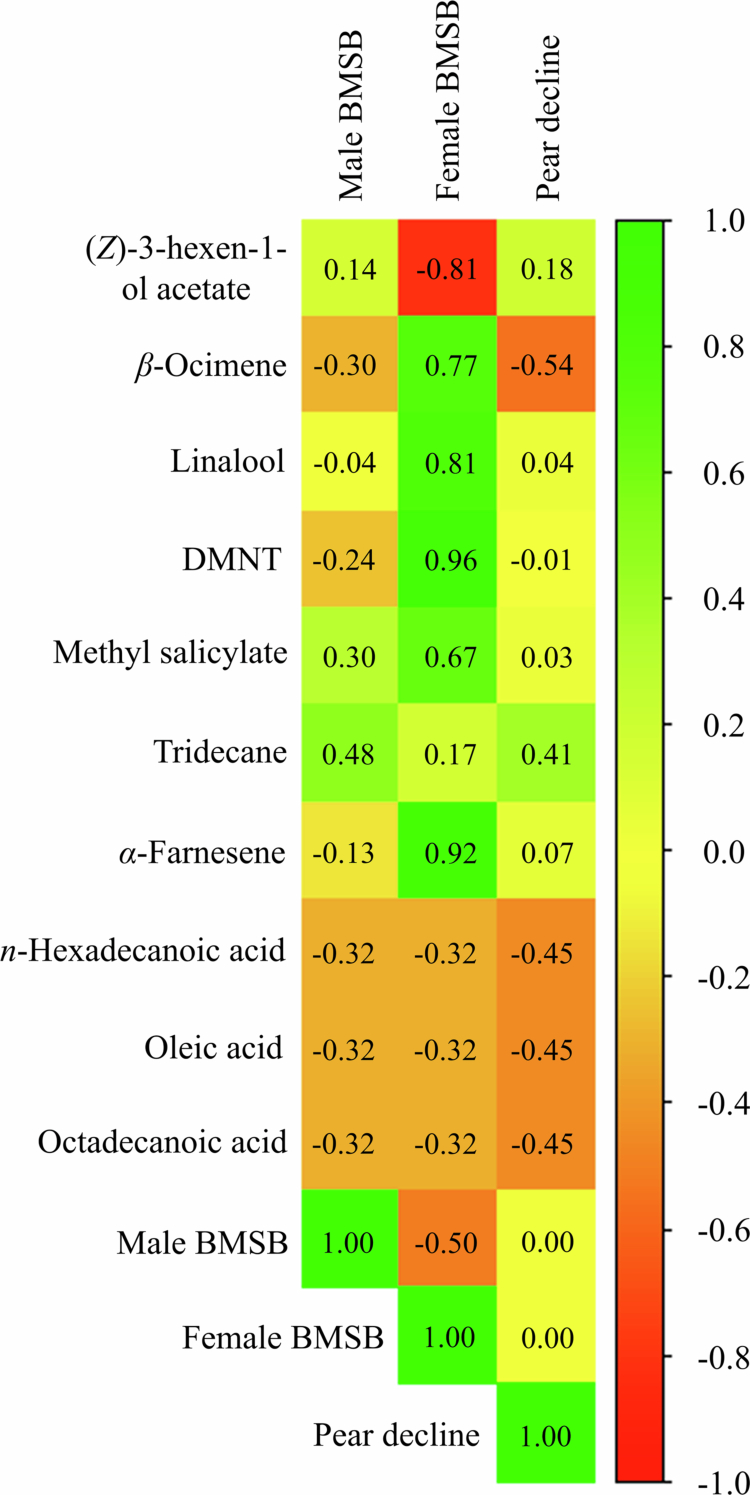
The correlogram represents the correlations between volatile compounds emitted by pear trees, BMSB feeding (male or female), and phytoplasma infection (PD). Positive correlations are displayed in green and negative correlations in red.

Overall, female BMSB feeding induced the most consistent and significant increases in pear VOC emissions, whereas PD infection alone resulted in comparatively minor changes. Moreover, positive correlations were found between female BMSB and *β*-ocimene (*r* = 0.77), linalool (*r* = 0.81), DMNT (*r* = 0.96), MeSA (*r* = 0.67), and *α*-farnesene (*r* = 0.92), whereas female BMSB had a negative correlation with (*Z*)-3-hexen-1-ol acetate (*r* = −0.81) (Figure 6). Besides, both male and female BMSB and PD phytoplasma had a negative correlation with octadecanoic acid, *n*-hexadecanoic acid, and oleic acid. Pear decline showed a negative correlation with *β*-ocimene (*r* = −0.54). The Pearson's correlation of male BMSB alone with tridecane (*r* = 0.48) was positive but not significant (adj. *p* > 0.5), whereas a significant emission was found in response to male BMSB in combination with PD-phytoplasma (adj. *p* = 0.002) (Table S5).

## 4. Discussion

Volatiles play a crucial role in plant communication, enabling plants to respond to and interact with their environment.^38–40^ Therefore, understanding how pest organisms of different pest guilds shape VOC emission is essential for clarifying their ecological roles, their mutual influences during co-infestation, and the consequences for plant-insect and plant-plant interactions.

In our study, the phytoplasma infection induced a pathway-specific VOC response in apple but not in pear trees. Apple trees infected with “*Ca*. P. mali” showed a significant increase in MeSA, a well-established SA-related defense volatile. This finding aligns with earlier work demonstrating that AP phytoplasma infection induces SA, jasmonic acid-isoleucine, and abscisic acid accumulation in apple with the virulent strain 12/93,^29^ and induced MeSA emission.^41^ In contrast to previous studies, we did not detect any significant increase in ethyl benzoate,^41^ or *β*-caryophyllene in phytoplasma-infected apple trees, the latter contributing to increased vector attraction of the vector insect *C. picta*.^42^ ^,^ ^43^ The absence of such responses in our material may reflect variability among phytoplasma strains, host genotypes, physiological states, or phenological stages of the sampled trees. Controversy to our results from apple, no phytoplasma-specific induction of SA-related VOCs was detectable in pear trees infected with “*Ca.* P. pyri”. This observation is consistent with Gallinger et al.,^29^ who found no measurable changes in SA levels in pear trees infected with PD-phytoplasma, suggesting either weaker virulence, host-specific tolerance, or an age-dependent response in pears.

Interestingly, in pear trees, the feeding activity of BMSB (both male and female) induced MeSA emissions but not in apple trees. Methyl salicylate is known to affect both direct and indirect plant defense responses to herbivore insects, such as aphids,^44^ and spider mites.^45^ Correlation analyzes of MeSA with AP indicated putative plant resistance to phytoplasmas in apple trees, as well as anti-herbivory defense mechanisms against BMSB in pear trees. This highlights that MeSA may have multiple mechanisms of action that can be effectively utilized in pest management.

Due to the piercing-sucking feeding mode of BMSB, we expected the insect to activate JA-, SA-, or mixed defense pathways.^5^ ^,^ ^6^ Indeed, Rondoni et al.^46^ showed that oviposition together with adult BMSB feeding induces an early and strong upregulation of JA-responsive genes (CPI, NAI1) in faba bean plants. In contrast, SA-related defenses (PR1 expression) are activated only later during prolonged nymphal feeding, following the decline of JA signaling and reflecting a negative crosstalk between JA and SA pathways.^46^ In terms of the specific responses of apple and pear trees to the BMSB, it was found that they released higher levels of linalool upon contact with the pest. In agreement with our results, it was found earlier that linalool was emitted at a higher rate in peach and the tree of heaven exposed to BMSB feeding and oviposition.^25^ Linalool is a terpenoid with demonstrated bioactivity against phytopathogens and herbivorous insects.^47^

Just like linalool, the homoterpene DMNT was specifically induced in response to BMSB exposure, but not to phytoplasma infections. In pears, linalool as well as DMNT were significantly induced by feeding of female BMSB (independent of a phytoplasma infection of the pear trees). Williams et al.^26^ reported that maize seedlings infested by *Nezara viridula* females emitted higher amounts of linalool than those infested by adult males, possibly due to increased feeding activity by females. DMNT has been reported as a key plant volatile in response to herbivore attack.^4^ ^,^ ^48^ DMNT can induce the accumulation of jasmonic acid in stems and leaves of herbivore-attacked plants, for example in sweet potato plants,^49^ which promotes the resistance of neighboring intact plants to herbivorous insects.^50^ Linalool, DMNT, and farnesene originate from the terpenoid biosynthesis pathway, which branches from the universal isoprenoid precursors. Nerolidol synthase 1 can react with both geranyl diphosphate and farnesyl pyrophosphate (FPP) to produce linalool or nerolidol, respectively.^51^ Nerolidol is the precursor of DMNT. Further, farnesene is a sesquiterpene hydrocarbon, also produced directly from FPP by farnesene synthase. Thus, monoterpenes and sesquiterpenes are closely linked: they share upstream precursors and often respond similarly to JA-regulated herbivore-induced signaling. It is notable that bioactive compounds are often related to each other in their biosynthesis, leading to co-regulation of their accumulation in plant tissues.^52^

In our study, the content of *α*-farnesene increased in pear trees exposed to mainly female BMSB, whilst phytoplasma-infected apple trees exposed to male BMSB induced the release of this volatile. Wang et al.^53^ reported that tea plants emitted *α*‑farnesene as part of their herbivore-induced volatile pattern when attacked by chewing and piercing-sucking insects. It is also noted that herbivore attacks activated jasmonic acid formation. Jasmonic acid significantly enhanced the expression level of *α*-farnesene synthase in *Camellia sinensis*, which led to the biosynthesis of *α*-farnesene.^53^

In the present study, the emission of (*Z*)-3-hexen-1-ol acetate decreased in response to BMSB feeding and AP infection in apple trees. (*Z*)-3-hexen-1-ol acetate is derived from C6-aldehydes as (*Z*)-3-Hexenal and *n*-Hexenal via the lipoxygenase (LOX) pathway from linolenic acid.^54^ Some stresses might activate or even suppress LOX activity. In accordance with our finding, (*Z*)-3-hexen-1-ol acetate was emitted at the lowest concentration from peach trees during BMSB feeding and oviposition, whereas it was induced from tree of heaven.^25^ (*Z*)-3-hexen-1-ol acetate not only appears to be an attractive compound for both beneficial and harmful insects,^55^ but this VOC also mediates the attraction of herbivores' natural enemies. It is possible that both apple and pear trees decreased the emission of (*Z*)-3-hexen-1-ol acetate to reduce the pressure of BMSB, as well as to avoid attracting phytoplasma vector insects.

According to our results, the content of tridecane highly increased solely in trees exposed to BMSB males. Although tridecane was found in plant species, it is the dominant VOC released by adult BMSB,^32^ which its emission increases with bug aggregation or density, acting as both a defensive and aggregation pheromone.^56^ Hence, it is assumed that increased levels of tridecane in host plant samples infested by male BMSB may be linked to stink bug frass, which could contain residual VOCs that leave distinct odor traces on the plants. Regarding the sex-specific volatile emission, our results showed that host plant volatile induction may be dependent on the biological sex of the BMSB feeding. For instance, female feeding predominantly stimulated the emission of plant-derived defense volatiles (linalool and DMNT), whereas male-exposed samples were characterized by higher levels of insect-derived defensive compounds (tridecane). This divergence suggests that while some plant defensive pathways are activated indiscriminately by general stink bug feeding, others are uniquely modulated by female-specific behaviors, such as oviposition,^46^ or sex-distinct salivary elicitors.^26^

Furthermore, we found three compounds in healthy pear trees, including *n*-hexadecanoic acid, octadecanoic acid, and oleic acid, which were not detected in phytoplasma-infected or BMSB-infested trees. In this study, the reduced oleic acid amount maybe linked to the increased MeSA levels. In soybeans, when the synthesis of oleic acid was silenced, plants induced constitutive defense signaling by salicylic acid accumulation and exhibited enhanced resistance to multiple pathogens.^57^ Although our study does not establish a causal relationship, the concurrent decrease in oleic acid and rise in MeSA suggests that BMSB feeding may alter volatile biosynthetic routes, shifting metabolic fluxes toward SA-related defense volatiles. Such modulation of VOC production could reflect a plant defense adjustment that potentially leads to reduced herbivore damage.

However, until now, it was a lack of information on the effect of BMSB male and female on volatile emissions of host plant. Besides, the details of the chemical ecology of the BMSB-pathogens-plant interactions are not completely known. For example, the status of BMSB as a vector of phytoplasmas is unknown, whereas *Nezara viridula*, another Pentatomidae species, has been shown to have the capacity to transmit the gram-negative bacterium *Pantoea agglomerans* into cotton bolls.^58^ Hence, further studies are now required to gain deeper insight into the role of VOCs released by plants in response to BMSB feeding and phytoplasma pathogens to determine the individual, synergistic, and antagonistic impacts of induced VOCs.

In conclusion, the distinct volatile profiles emitted by apple and pear trees under single or dual pest pressure demonstrated that host plants activate specialized chemical responses to piercing-sucking herbivory and phloem bacterial infections. In this study, pome fruit trees infested with BMSB or infected with phytoplasma released characteristic individual volatiles, including linalool, DMNT, and methyl salicylate, which differentiated the treatment groups. Furthermore, the clear separation of uninfested control plants within the shared chamber suggests that background ambient volatiles did not induce confounding systemic shifts in neighboring unexposed trees. Rather than acting as broad ecological signals, these VOCs may serve as pest-specific indicators of specific stress. These distinct chemical footprints provide a useful framework for the development of non-invasive diagnostic tools, such as electronic nose sensors, to detect, differentiate, and monitor invasive stink bug infestations and phytoplasma outbreaks within integrated pest management strategies.

## Supplementary Material

Supp_Table 2_5.docx Supp_Table 2_5.docx

Supplementary Table S1A_B.xlsx Supplementary Table S1A_B.xlsx

## Data Availability

The data generated and/or analyzed during this study are available from the corresponding author upon reasonable request.
