# Supplementary material for: Dual biotic stressors shape volatile organic compound emission patterns in pome fruit trees
Source: Plant Signal Behav. 2026 Jul 9;21(1):2700902. doi: 10.1080/15592324.2026.2700902 (PMC13353780; doi:10.1080/15592324.2026.2700902)
Supplement: Supp_Table 2_5.docx [file KPSB_A_2700902_SM3018.docx]

This document provides supplementary material supporting the results presented in the main paper.

Table S2. Summary of Random Forest (RF) classification outcomes for the separation of VOC profiles among apple tree treatments.

| Confusion matrix | Apple proliferation infection | | | Control and BMSB infested apple trees | | | Class error |
| --- | --- | --- | --- | --- | --- | --- | --- |
|  | AP | AP + Female BMSB | AP + Male BMSB | Control | Female BMSB | Male BMSB |  |
| AP | 4 | 0 | 1 | 0 | 0 | 0 | 0.2 |
| AP + Female BMSB | 0 | 3 | 1 | 0 | 0 | 0 | 0.25 |
| AP + Male BMSB | 1 | 0 | 4 | 0 | 0 | 0 | 0.2 |
| Control | 1 | 0 | 0 | 4 | 0 | 0 | 0.2 |
| Female BMSB | 0 | 0 | 0 | 0 | 3 | 2 | 0.4 |
| Male BMSB | 0 | 0 | 1 | 0 | 2 | 3 | 0.4 |
| No. of variables tried at each split: 8 | | | | | | | |
| OOB error rate | 24.14% | | | | | | |
| OOB error rate: left | 37.93% | | | | | | |
| OOB error rate: right | 17.24% | | | | | | |

*Note. AP = phytoplasma-infected trees; male BMSB = male H. halys infestation; female BMSB = female H. halys infestation; dual infestations = AP + male BMSB and AP + female BMSB.*

Table S3. Summary of ANOVA results (*p*-values) for differences in individual VOC emission patterns across multiple infestation treatments in apple trees.

| VOC compounds | Control | Control | Control | Control | Control |
| --- | --- | --- | --- | --- | --- |
|  | Male BMSB | Female BMSB | AP | AP + Male BMSB | AP + Female BMSB |
| (*Z*)-3-hexen-1-ol acetate | 0.001265 | 0.012099 | 0.1005 | 0.000829 | 0.044728 |
| linalool | 0.044784 | 0.001478 | 0.396578 | 0.023063 | 0.040629 |
| DMNT | 0.005177 | 0.060996 | 0.996168 | 0.199268 | 0.957313 |
| methyl salicylate | 0.992171 | 0.999572 | 0.022992 | 0.525497 | 0.680515 |
| tridecane | 0.015038 | 0.999955 | 0.994018 | 0.00098 | 0.622347 |
| geranylaceton | 0.011297 | 0.111521 | 0.26033 | 0.281533 | 0.011623 |
| α-farnesene | 0.999997 | 0.999999 | 0.960536 | 0.054145 | 0.999885 |

*Note. Significant differences are displayed in green. AP = phytoplasma-infected trees; male BMSB = male H. halys infestation; female BMSB = female H. halys infestation; dual infestations = AP + male BMSB and AP + female BMSB.*

Table S4. Summary of Random Forest (RF) classification outcomes for the separation of VOC profiles among pear tree treatments.

| Confusion matrix | Pear decline infection | | | Control and BMSB infested apple trees | | |  |
| --- | --- | --- | --- | --- | --- | --- | --- |
|  | PD | PD + Female BMSB | PD + Male BMSB | Control | Female BMSB | Male BMSB | Class error |
| PD | 5 | 0 | 0 | 0 | 0 | 0 | 0 |
| PD + Female BMSB | 0 | 4 | 1 | 0 | 0 | 0 | 0.2 |
| PD + Male BMSB | 0 | 1 | 4 | 0 | 0 | 0 | 0.2 |
| Control | 0 | 0 | 0 | 5 | 0 | 0 | 0 |
| Female BMSB | 0 | 0 | 1 | 0 | 4 | 0 | 0.2 |
| Male BMSB | 0 | 0 | 1 | 0 | 0 | 4 | 0.2 |
| No. of variables tried at each split: 8 | | | | | | | |
| OOB error rate | 6.67% | | | | | | |
| OOB error rate: left | 13.33% | | | | | | |
| OOB error rate: right | 10.00% | | | | | | |

*Note. PD = phytoplasma-infected trees; male BMSB = male H. halys infestation; female BMSB = female H. halys infestation; dual infestations = PD + male BMSB and PD + female BMSB.*

Table S5. Summary of ANOVA results (*p*-values) for differences in individual VOC emission patterns across multiple infestation treatments in pear trees.

|  | Control | Control | Control | Control | Control |
| --- | --- | --- | --- | --- | --- |
|  | Male BMSB | Female BMSB | PD | PD + Male BMSB | PD + Female BMSB |
| (*Z*)-3-Hexen-1-ol acetate | 0.999405 | 0.683797 | 0.283251 | 0.999999 | 0.479829 |
| *ß*-Ocimene | 1 | 0.000173 | 0.298873 | 0.913575 | 0.967026 |
| Linalool | 0.12851 | 0.008068 | 0.611865 | 0.558395 | 0.017537 |
| DMNT | 0.910347 | 0.023866 | 0.999283 | 0.962738 | 0.008971 |
| Methyl salicylate | 0.088882 | 0.004195 | 0.999999 | 0.021186 | 0.006893 |
| Tridecane | 0.995714 | 0.457059 | 1 | 0.002407 | 0.535225 |
| *α*-Farnesene | 0.914412 | 0.043023 | 0.999908 | 0.706563 | 0.017917 |
| *n*-Hexadecanoic acid | 0.002029 | 0.002029 | 0.002029 | 0.002029 | 0.002029 |
| Oleic acid | 8.50E-06 | 8.50E-06 | 8.50E-06 | 8.50E-06 | 8.50E-06 |
| Octadecanoic acid | 4.00E-07 | 4.00E-07 | 4.00E-07 | 4.00E-07 | 4.00E-07 |

*Note. Significant differences are displayed in green. PD = phytoplasma-infected trees; male BMSB = male H. halys infestation; female BMSB = female H. halys infestation; dual infestations = PD + male BMSB and PD + female BMSB.*
